# Association Between Gestational Age and Academic Achievement of Children Born at Term

**DOI:** 10.1001/jamanetworkopen.2023.26451

**Published:** 2023-07-31

**Authors:** George L. Wehby

**Affiliations:** 1 Department of Health Management and Policy, University of Iowa, Iowa City; 2 Department of Economics, University of Iowa, Iowa City; 3 Department of Preventive and Community Dentistry, University of Iowa, Iowa City; 4 Public Policy Center, University of Iowa, Iowa City; 5 National Bureau of Economic Research, Cambridge, Massachusetts

## Abstract

**Importance:**

Differences in academic achievement by gestational age of children born at term, especially at 39 to 41 weeks, are not well understood.

**Objective:**

To examine differences in academic achievement among children born between 37 and 41 weeks’ gestational age.

**Design, Setting, and Participants:**

This retrospective cohort study linked birth certificates of children born in Iowa from 1989 to 2009 with school test scores for grades 2 to 11 from 2017 to 2018. Statistical analysis was performed from January to March 2023.

**Exposures:**

Gestational age at 37, 38, 39, and 41 weeks vs 40 weeks from clinical or obstetric and calendar measures.

**Main Outcomes and Measures:**

Outcomes were scores in national percentile rankings (NPRs) on standardized school tests in math and reading. Covariates included demographic and prenatal risk factors.

**Results:**

The sample included 536 996 children (50.7% male children and 49.3% female children) with math scores (3 576 045 child-grade observations; 6.6%, 15.7%, 28.6%, 35.5%, and 13.7% born at 37, 38, 39, 40, and 41 weeks, respectively) and 537 078 children with reading scores (3 590 408 child-grade observations). Score differences for those born at 39 vs 40 weeks were −0.028 NPRs (95% CI, −0.18 to 0.12 NPRs) for math and 0.085 NPRs (95% CI, −0.067 to 0.24 NPRs) for reading using the clinical or obstetric measure and 0.03 NPRs (95% CI, −0.14 to 0.20 NPRs) for math and 0.13 NPRs (95% CI, −0.042 to 0.31 NPRs) for reading using the calendar measure. With the clinical or obstetric measure, score differences between those born at 41 and 40 weeks were 0.19 NPRs (95% CI, −0.0052 to 0.38 NPRs) for math and 0.098 NPRs (95% CI, −0.096 to 0.29 NPRs) for reading. With the calendar measure, score differences for those born at 41 weeks were −0.22 NPRs (95% CI, −0.43 to −0.013 NPRs) for math and −0.28 NPRs (95% CI, −0.49 to −0.074 NPRs) for reading. With the clinical or obstetric measure, score differences between those born at 37 and 38 weeks vs 40 weeks were −0.59 NPRs (95% CI, −0.84 to −0.33 NPRs) and −0.44 NPRs (95% CI, −0.62 to −0.26 NPRs), respectively, for math, and −0.066 NPRs (95% CI, −0.32 to 0.19 NPRs) and −0.19 NPRs (95% CI, −0.37 to 0.0038 NPRs), respectively, for reading.

**Conclusions and Relevance:**

This study suggests that there is no evidence of a difference in math and reading scores over grades 2 to 11 among children born between 39 and 40 weeks’ gestation, and overall no evidence of better scores among those born at 41 weeks’ gestation compared with 40 weeks’ gestation. The results can further inform decisions on delivery timing at term birth by offering insights into long-term associations of delivery timing with cognitive development and school achievement.

## Key Points

**Question:**

What is the difference in school math and reading test scores by gestational age for children born at term?

**Findings:**

In this population-based cohort study of more than 500 000 children, there was no difference in school achievement between children born at 39 vs 40 weeks, and no evidence of better test scores between children born at 41 weeks vs 40 weeks. Scores were generally lower for children born at 37 to 38 weeks vs 40 weeks.

**Meaning:**

Those making decisions about delivery timing at term can consider these new insights about differences in school achievement when weighing short-term and long-term benefits and risks.

## Teaser

This cohort study of more than 500 000 children examines differences in academic achievement among children born between 37 and 41 weeks’ gestational age.

## Introduction

It is well known that children born preterm have a higher risk of perinatal complications and poorer developmental outcomes, including lower academic achievement, during childhood.^1,2,3^ However, for children who are born at term, the association between gestational age and subsequent developmental outcomes, such as cognition and academic achievement, is complex. During the period of 37 to 41 gestational weeks, 37 to 38 weeks is considered early term, 39 to 40 weeks is considered full term, and 41 weeks are considered late term.^4^ Within this range, neonatal outcomes are generally worse at 37 to 38 weeks,^5^ and 39 to 40 weeks is generally considered an optimal period for minimizing perinatal complications.^6^

Current obstetric guidelines recommend that pregnant women at low risk and without a clinical indication for labor induction do not undergo induction within 37 to 38 weeks to avoid perinatal complications, including respiratory problems.^7^ At the same time, delivery is recommended no later than 41 weeks for women who reached this time, to avoid perinatal complications.^6^ Within the 39- to 40-week range, however, there is less certainty whether an earlier delivery is optimal or not. Evidence from a randomized clinical trial indicated a lower risk of cesarean delivery with labor induction at 39 weeks vs expectant management, although there was no statistically significant difference in a measure of perinatal complications.^8^

Even though perinatal and maternal health outcomes are essential to these clinical guidelines, there is also increasing evidence and attention to the implications of differences in gestational age within this period for subsequent child development outcomes, including cognitive development and school achievement. However, few studies with data from population-based samples have addressed this question; these studies report lower school performance in the form of test scores or teacher rankings for children born at 37 to 38 weeks compared with 39 to 40 weeks.^9,10,11^ Two of these studies, one using birth certificate data linked to school test scores in Florida for more than 1 million children^9^ and another using data for approximately 1400 children from the Fragile Families and Child Well-being study,^10^ report better school outcomes for children born at 41 weeks compared with 39 to 40 weeks. However, another study using birth certificate data linked to school test scores for more than 100 000 children in New York City found a small and statistically nonsignificant difference between reading and math scores for children born between 40 and 41 weeks, although the study reported slightly higher math scores among those born at 39 weeks relative to 41 weeks.^11^ Also, that study reported lower reading scores for those born at 39 weeks than those born at 40 weeks, although the difference was marginally significant (*P* = .06).

This study examines the association between gestational age within 37 to 41 weeks and school achievement using unique population-based data of birth certificates linked to standardized school test scores in Iowa. The study addresses 2 gaps in the literature. First, little is known about the difference in outcomes between children born at 39 weeks gestational age and children born at 40 weeks’ gestational age because these 2 age groups have mostly been combined in the literature. Considering the evidence on decreases in cesarean delivery risk among nulliparous women at low risk whose delivery was induced at 39 weeks compared with expectant management^8^ and the variation in clinical decisions around the timing of delivery within this range, further evidence is needed on the long-term cognitive and developmental outcomes of children delivered at 39 or 40 weeks. This study separates and compares these 2 gestational ages. Another contribution of this study is examining the differences in school test scores grade by grade from grades 2 to 11 to understand the timing of any differences in outcomes and whether they are stable, grow, or decrease over time. Previous studies focused on term birth have either pooled different grades^9^ or focused on 1 grade or age (eg, third grade or 9 years of age^10,11^). Evaluating how gestational age within the term range is associated with achievement over the child’s life can reveal whether there are any sensitive periods when differences emerge or accelerate and vice versa, offering important knowledge about windows of opportunity to intervene with greater impact to address gaps.

## Methods

### Data Sources

The data for this study come from 2 sources: the birth certificates of children born in the state of Iowa between 1989 and 2009 obtained from the Iowa Department of Public Health and student scores on the standardized school tests for children in grades 2 through 11 and available through school year 2017 to 2018 obtained from the Iowa Testing Programs. The birth year period for this study began in 1989 because this is the first year when the clinical measure of gestational age was reported in the birth certificates and ended in 2009 because a very small fraction of children born after that year had complete school test scores by 2017 to 2018. The 2 data sources were independently matched by child’s name and date of birth. The sample was limited to singleton births given the association of multiple births with lower gestational age. Of 683 732 singleton births between 37 and 41 gestational weeks in the birth certificates, 553 459 (80.9%) were matched to at least 1 school test score record. Among those, 536 996 children had at least 1 math test score (a total of 3 576 045 child-grade observations with math test scores) and had complete data on all model variables, and 537 078 children had at least 1 reading test score (3 590 408 child-grade observations) and had complete data on all model variables. For the grade-specific analyses, the sample size ranged from 216 785 to 480 805 observations depending on grade (smallest sample for grade 2 and largest sample for grade 3) using the clinical or obstetric gestational age measure and from 180 157 to 398 811 observations using the calendar gestational age measure. Data linkages were completed in a way so that the researchers only had access to deidentified data and approved by the University of Iowa institutional review board with an informed consent waiver. This study followed the Strengthening the Reporting of Observational Studies in Epidemiology (STROBE) reporting guideline for observational studies.

### Measures

The study compares children born at the gestational ages of 37, 38, 39, and 41 weeks each with those born at 40 weeks. Two measures of gestational age are evaluated in separate models. The primary measure is based on the clinical estimate (in years 1989-2002) and the obstetric estimate (in birth years 2003-2009) recorded in birth certificates, which is also used to select the analytical sample; we refer to this as the clinical or obstetric measure. Switching to the obstetric estimate in birth certificates came with more detailed instructions to the birth attendant, including not basing the estimate on neonatal examinations, although otherwise the measures are generally considered to be comparable.^12^ Evidence suggests that the obstetric estimate is a valid measure.^13^ A secondary measure is the calendar estimate, based on the delivery date minus the date of the last menstrual period before pregnancy. With that measure available in days, the following gestational age ranges are compared with 40 weeks and 0 to 6 days: 37 weeks and 0 to 6 days, 38 weeks and 0 to 6 days, 39 weeks and 0 to 6 days, and 41 weeks and 0 to 6 days. This measure is evaluated because it may be considered in clinical decision-making and because it provides gestational age in days. It is well known, however, that this measure does not correlate well with the clinical or obstetric estimate, especially at late term.^12,14^

The school tests are based on the Iowa Tests of Basic Skills, Iowa Tests of Educational Development, and Iowa Assessments.^15^ Both public and private schools routinely administer these tests, and scores on these tests have been previously associated with conceptually relevant health and policy factors, further supporting their validity.^16,17,18,19,20,21,22^ This study focuses on math and reading tests, the 2 most commonly tested subjects. The 2 outcome measures are standardized test scores on math and reading, shown in national percentile rankings (NPRs), so the differences in scores across gestational ages can be directly interpreted as differences in percentile rankings relative to a national sample.

### Statistical Analysis

Statistical analysis was performed from January to March 2023. To estimate the differences in school test scores by gestational age, the test scores are regressed on indicators for gestational ages of 37, 38, 39, and 41 weeks, with 40 weeks as the omitted or reference category. The regression model adjusts for multiple covariates from the birth certificate data, including maternal age (flexibly using binary indicators for age in years), marital status (married or not), race and ethnicity (an indicator for whether the mother is White or all other races [American Indian or Alaskan Native, Asian, Black, or Pacific Islander mothers were combined for “other race” because of the low frequency of these races], and another indicator for whether the mother is Hispanic or not), and educational level (less than high school, high school, some college, and college graduate). An indicator for race and another for ethnicity were included to further capture socioeconomic factors that are related to school achievement. Additional covariates included child’s sex (male or female) and fixed effects for month of birth and year of birth from the birth certificate data, as well as fixed effects for school grade (for models pooling by grade) and semester and year of testing from the school test data. Also included are an indicator for whether the child has a reported congenital anomaly in the birth certificate, an indicator for any maternal health problem (such as gestational diabetes, gestational hypertension, or previous preterm birth), the number of previous live births the mother has had, whether the mother smoked during pregnancy, number of prenatal visits, and school district fixed effects. In addition to this first regression, another model adds as controls one indicator for whether the birth was induced and another indicator for cesarean delivery. These 2 covariates are added in a separate model because they are potentially influenced by gestational age. In addition to the model pooling by grade level, a separate regression is estimated for each grade level to evaluate how differences in test scores by gestational age change over the child’s age (grade). The regression is estimated by ordinary least squares; in the model pooling by grade level with repeated observations for the same child (repeated grade levels), SEs are clustered at the child level. All *P* values were from 2-sided tests, and results were deemed statistically significant at *P* < .05. Data analysis was performed using Stata, version 15.1 (StataCorp LP).^23^

## Results

### Sample Description

The sample included 536 996 children with math scores (3 576 045 child-grade observations; 6.6%, 15.7%, 28.6%, 35.5%, and 13.7% born at 37, 38, 39, 40, and 41 weeks, respectively, and 50.7% for male children and 49.3% for female children) and 537 078 children with reading scores (3 590 408 child-grade observations) (Table 1). A total of 38.0% of observations were for mothers who had no prior live births, 95.8% were for White mothers, 76.4% were for married mothers, 11.5% were for mothers with less than high school education, 32.4% were for mothers with high school education, and 18.2% were for mothers who smoked during pregnancy. Mean (SD) scores in the analytical samples were 62.4 (26.7) NPRs for math and 60.8 (26.9) NPRs for reading.

**Table 1.** Analytical Sample Description

Variable	Value^a^
Child-grade observations for math scores, No.	3 576 045
Gestational age (clinical or obstetric measure) in weeks, %	
37	6.6
38	15.7
39	28.6
40	35.5
41	13.7
School test scores, mean (SD), NPRs	
Math	62.4 (26.7)
Reading comprehension	60.8 (26.9)
Demographic, socioeconomic, and infant health variables	
Child’s sex, %	
Female	49.3
Male	50.7
No. of prior live births, %	
0	38.0
1	35.0
2	18.2
3	6.1
≥4	2.7
Maternal age at child’s birth (years), %	
<20	8.6
20-24	25.0
25-29	33.2
≥30	33.2
Maternal race and ethnicity, %	
White	95.8
All other races and ethnicities^b^	4.2
Maternal Hispanic ethnicity, %	
Yes	3.5
No	96.5
Maternal marital status at child’s birth, %	
Married	76.4
Not married	23.6
Maternal educational level at child’s birth, %	
Less than high school	11.5
High school	32.4
Some college	30.7
College graduate	25.4
Prenatal visits, mean (SD), No.	12.1 (3.0)
Maternal smoking during pregnancy, %	
Yes	18.2
No	81.8
Child has congenital anomalies, %	
Yes	0.2
No	99.8
Mother had health complications, %	
Yes	5.1
No	94.9
Labor induction, %	
Yes	20.2
No	79.8
Cesarean delivery, %	
Yes	20.4
No	79.6

Abbreviation: NPR, national percentile ranking.

^a^ Summary statistics for math scores and household covariates are for the analytical sample with complete data on those variables and included in regressions for math scores (3 576 045 child-grade observations). The mean (SD) for the reading test score is based on the sample with complete data on that measure and household covariates and included in regressions for reading scores (3 590 408 child-grade observations). Summary statistics (mean [SD] values or percentages) are for math and reading test scores, gestational age, and household characteristics for the sample pooled across grades.

^b^ Included American Indian or Alaskan Native, Asian, Black, or Pacific Islander; they were combined into 1 category because of the low frequency of these races and ethnicities in the sample.

### Score Differences Pooling by Grade

Table 2 shows differences in math and reading scores by gestational age based on the clinical or obstetric measure, pooled across grades 2 through 11. Table 3 shows these estimates for the calendar measure of gestational age. The results are from separate models for clinical or obstetric and calendar gestational age measures; each model was run with and then again without controlling for induced labor and cesarean delivery, which overall has little association with the estimates. Score differences for children born at 39 weeks compared with those born at 40 weeks were very small and statistically insignificant; in the fully adjusted model and based on the clinical or obstetric measure, the difference was −0.028 NPRs (95% CI, −0.18 to 0.12 NPRs) for math and 0.085 NPRs (95% CI, −0.067 to 0.24 NPRs) for reading (Table 2); with the calendar measure, the difference was 0.03 NPRs (95% CI, −0.14 to 0.20 NPRs) for math and 0.13 NPRs (95% CI, −0.042 to 0.31 NPRs) for reading (Table 3). There was no evidence of higher scores for children born at 41 vs 40 gestational weeks; based on the clinical or obstetric gestational measure, there was no discernable difference in test scores between the 2 gestational ages: 0.19 NPRs (95% CI, −0.0052 to 0.38 NPRs) for math and 0.098 NPRs (95% CI, −0.096 to 0.29 NPRs) for reading (Table 2). In contrast, scores were lower for children born at 41 weeks based on calendar gestational age: –0.22 NPRs (95% CI, −0.43 to −0.013 NPRs) for math and –0.28 NPRs (95% CI, −0.49 to −0.074 NPRs) for reading (Table 3). Consistent with the literature, math scores were lower for children born at 37 and 38 gestational weeks relative to those born at 40 weeks; in the fully adjusted model and based on the clinical or obstetric measure, math scores were –0.59 NPRs (95% CI, −0.84 to −0.33 NPRs) and –0.44 NPRs (95% CI, −0.62 to −0.26 NPRs) for those born at 37 and 38 weeks, respectively, relative to those born at 40 weeks (Table 2). Reading scores were −0.066 NPRs (95% CI, −0.32 to 0.19 NPRs) and −0.19 NPRs (95% CI, −0.37 to 0.0038 NPRs) for those born at 37 and 38 weeks, respectively, relative to those born at 40 weeks. Differences in math scores were larger for those born at 37 weeks but similar for those born at 38 weeks based on the calendar compared with the clinical or obstetric gestational age measure. In contrast, reading differences between children born at 37 to 38 weeks and those born at 40 weeks were less pronounced than math differences.

**Table 2.** Regression Estimates of Differences in Math and Reading Scores by Gestational Age Based on the Clinical or Obstetric Measure^a^

Variable	Math test scores, NPRs (95% CI)		Reading test scores, NPRs (95% CI)	
	No adjustment for labor induction and cesarean delivery	Adjustment for labor induction and cesarean delivery	No adjustment for labor induction and cesarean delivery	Adjustment for labor induction and cesarean delivery
Gestational age, wk				
37	−0.67 (−0.93 to −0.42)	−0.59 (−0.84 to −0.33)	−0.14 (−0.40 to 0.11)	−0.066 (−0.32 to 0.19)
38	−0.53 (−0.71 to −0.35)	−0.44 (−0.62 to −0.26)	−0.27 (−0.46 to −0.088)	−0.19 (−0.37 to −0.0038)
39	−0.13 (−0.28 to 0.021)	−0.028 (−0.18 to 0.12)	−0.0056 (−0.16 to 0.15)	0.085 (−0.067 to 0.24)
40	[Reference]	[Reference]	[Reference]	[Reference]
41	0.13 (−0.065 to 0.32)	0.19 (−0.0052 to 0.38)	0.046 (−0.15 to 0.24)	0.098 (−0.096 to 0.29)
Child-grade observations, No.	3 576 045	3 576 045	3 590 408	3 590 408

Abbreviation: NPR, national percentile ranking.

^a^ The regressions are estimated for the sample pooled across grades.

**Table 3.** Regression Estimates of Differences in Math and Reading Scores by Gestational Age Based on the Calendar Measure^a^

Variable	Math test scores, NPRs (95% CI)		Reading test scores, NPRs (95% CI)	
	No adjustment for labor induction and cesarean delivery	Adjustment for labor induction and cesarean delivery	No adjustment for labor induction and cesarean delivery	Adjustment for labor induction and cesarean delivery
Gestational age, wk				
37	−0.96 (−1.23 to −0.69)	−0.89 (−1.16 to −0.63)	−0.45 (−0.71 to −0.18)	−0.39 (−0.65 to −0.12)
38	−0.52 (−0.72 to −0.32)	−0.42 (−0.62 to −0.22)	−0.24 (−0.44 to −0.037)	−0.15 (−0.35 to 0.054)
39	−0.044 (−0.22 to 0.13)	0.03 (−0.14 to 0.20)	0.065 (−0.11 to 0.24)	0.13 (−0.042 to 0.31)
40	[Reference]	[Reference]	[Reference]	[Reference]
41	−0.27 (−0.48 to −0.063)	−0.22 (−0.43 to −0.013)	−0.33 (−0.54 to −0.12)	−0.28 (−0.49 to −0.074)
Child-grade observations, No.	2 936 877	2 936 877	2 948 312	2 948 312

Abbreviation: NPR, national percentile ranking.

^a^ The regressions are estimated for the sample pooled across grades.

### Grade-by-Grade Differences

**Figure 1.**
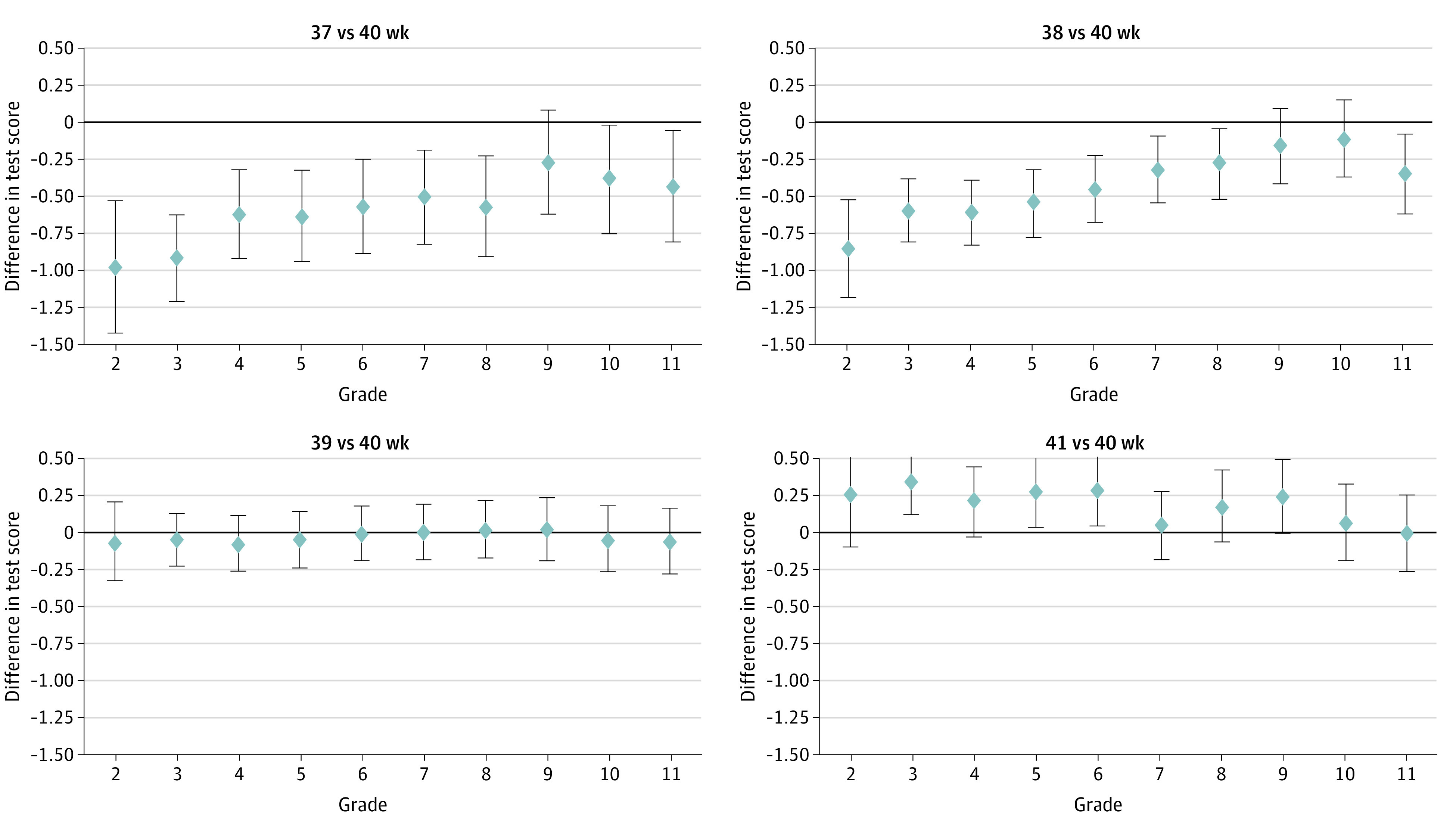
Regression Estimates of Differences in Math Scores by Gestational Age (Clinical or Obstetric Measure) and Grade The point estimates are from a separate regression for each grade that estimates the differences in math scores across gestational age groups (40 weeks as the reference group). The regression adjusts for all covariates, including labor induction and cesarean delivery. The differences in test scores are in national percentile rankings. The error bars around the point estimates indicate the 95% CIs.

**Figure 2.**
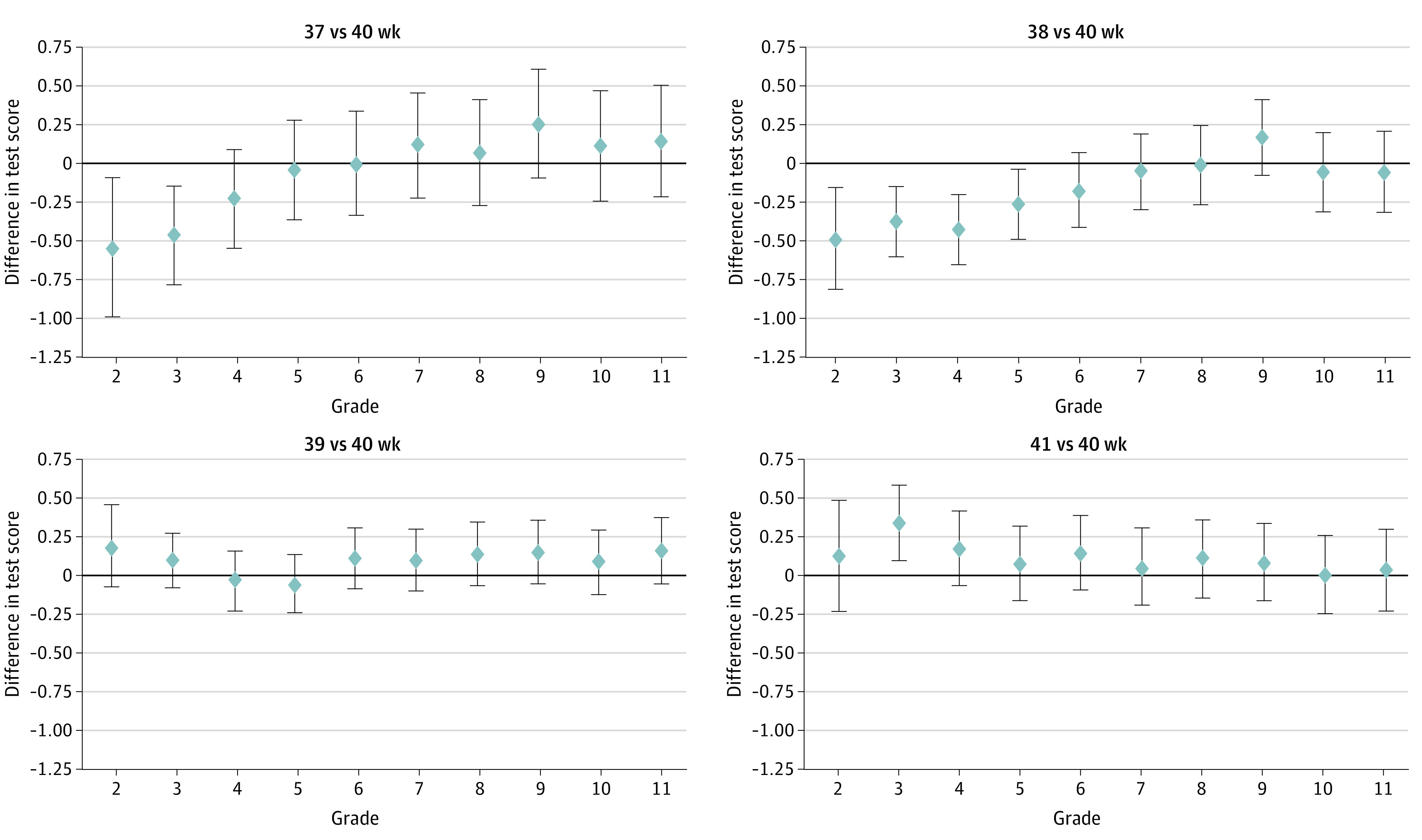
Regression Estimates of Differences in Reading Scores by Gestational Age (Clinical or Obstetric Measure) and Grade The point estimates are from a separate regression for each grade that estimates the differences in reading scores across gestational age groups (40 weeks as the reference group). The regression adjusts for all covariates, including labor induction and cesarean delivery. The differences in test scores are in national percentile rankings. The error bars around the point estimates indicate the 95% CIs.

Figure 1 and Figure 2 show grade-by-grade differences in math and reading scores, respectively, by the clinical or obstetric gestational age measure; the results are from the regression with the full set of control variables, including labor induction and cesarean delivery, estimated separately for each grade. There were no discernible differences in math and reading scores between children born at 39 gestational weeks and children born at 40 gestational weeks. Among children born at 41 gestational weeks compared with 40 gestational weeks, math test scores were higher in grades 3, 5, and 6, and reading scores were higher in grade 3, but there were no other statistically significant differences between the 2 gestational age groups at other grades, including grades 10 and 11, for which the differences were essentially null. For children born at gestational age 37 to 38 weeks compared with 40 gestational weeks, math scores were significantly lower for most grades, although there was a trend of differences decreasing at higher grades. In contrast, reading scores were only lower for grades 2 and 3 for children born at gestational age 37 weeks compared with 40 weeks and for grades 2 to 5 for those born at gestational age 38 weeks compared with 40 weeks. Above these grades, the reading score differences for children born at 37 to 38 weeks vs 40 weeks were small and no longer different from 0.

eFigures 1 and 2 in Supplement 1 show the grade-by-grade differences in math and reading scores, respectively, by calendar gestational age. The results for children born at 37, 38, and 39 gestational weeks vs 40 gestational weeks are largely consistent with those based on the clinical or obstetric gestational age measure. In contrast, for children born at 41 gestational weeks, scores are lower than those for children born at 40 weeks, with significant differences at grades 7, 8, and 11 for math and grades 5, 6, 7, and 9 for reading.

## Discussion

Based on a large population-based sample of children in Iowa, this study evaluated differences in scores on standardized math and reading tests by gestational age at term. The score differences between children born at 39 weeks and children born at 40 weeks were very small and statistically nonsignificant. In contrast to 2 prior studies,^9,10^ no evidence was found of higher scores for children born at 41 vs 40 gestational weeks based on the clinical or obstetric gestational measure; there is no discernable difference between the 2 gestational ages when averaging across grades and in most grades when analyzed separately. However, based on the calendar gestational age measure, math and reading scores were lower among those born at 41 weeks when averaging across grades and in some grades. The differences in the results between the clinical or obstetric measure and the calendar gestational age measure for children born at 41 weeks are likely due to the poor concordance between the 2 measures at late-term birth. Consistent with the literature, the study found lower math scores for children born at gestational age 37 to 38 weeks compared with 40 weeks. However, in contrast to the literature, it found a less pronounced difference in reading scores than in math scores between children born at 37 to 38 weeks and those born at 40 weeks. Furthermore, there appears to be attenuation of differences in math scores at higher grades between children born at 37 to 38 weeks and those born at 40 weeks, suggesting the potential for reducing these gaps over time, although more research is needed to directly investigate such associations. Finally, the estimated associations between gestational age and test scores did not meaningfully differ by whether labor induction and cesarean delivery were adjusted for or not, suggesting other mechanisms for these associations.

The findings offer new insights into the long-term associations of child cognitive development and school achievement with delivery timing at term. Specifically, the study indicates that there is no benefit to achievement associated with delivering at 40 weeks rather than 39 weeks. Considering the increased risk of cesarean delivery with expectant management at 39 weeks instead of labor induction reported in a randomized clinical trial of nulliparous women at low risk, and the evidence of no difference in school achievement between children born at 39 weeks and those born at 40 weeks,^8^ clinicians and expectant mothers can make more informed decisions weighing clinical and developmental benefits and risks to mothers and children associated with labor induction at 39 weeks vs expectant management. Another insight is the apparent lack of benefits to achievement associated with delivering at 41 weeks compared with 40 weeks. This evidence further supports current guidelines for delivering no later than 41 weeks^6^ and defining 41 weeks as late term.^4^ A third insight is that differences in achievement between children born at 37 to 38 weeks and those born at 40 weeks appear to attenuate as age (grade) increases and are relatively small for reading, suggesting the potential for school programs to reduce early child developmental differences between early-term and full-term births. Current guidelines for medically indicated early-term delivery emphasize considering maternal and infant risks from delivery vs waiting^24^; the current study sheds further light on long-term differences in achievement between early-term and full-term births that may also be useful for future guidelines.

### Strengths and Limitations

The study has several strengths, including that it is population-based with a large sample of children across all evaluated grades, that it includes a grade-by-grade analysis from grades 2 to 11, that it uses standardized school tests, and that data were available on multiple conceptually relevant covariates for mothers and infants. This study also has limitations. One weakness is the potential for confounding from clinical risk factors not sufficiently captured in the birth certificate data. Also, there is likely some measurement error in gestational age, especially in the calendar measure. Results are provided for the clinical or obstetric measure, which is considered more valid, and the calendar measure; results are broadly consistent, except among children born at 41 weeks, where the 2 measures are less concordant. Another limitation is generalizability to other more racially, ethnically, and socioeconomically diverse populations. Reexamining these questions with data sets from other states, possibly combined with electronic health record data when such linkages become possible, would help in addressing these issues. Furthermore, adding child health and development outcomes to randomized clinical trials that test changes to delivery timing management is also important to capture their effects on children’s development and school achievement. Examining longer-term socioeconomic outcomes, such as educational attainment and income, would also be important. Additionally, there were no data in this study on household investments in children and how investments may differ by early child health and development. Adding such data in future studies would be useful to better understand the dynamics between gestational age and related early and long-term health outcomes.

## Conclusions

This cohort study of more than 500 000 children found no evidence of a difference in math and reading scores over grades 2 to 11 between children born at 39 gestational weeks and those born at 40 gestational weeks, and overall no evidence of better scores among those born at 41 weeks than those born 40 weeks. These results can be useful to further weigh the long-term associations with children’s cognitive development and school achievement in decisions on delivery timing at term birth in addition to the other benefits and risks to mothers and infants.

## Notes

Supplement 1. **eFigure 1.** Regression Estimates of Differences in Math Scores by Gestational Age (Calendar Measure) and Grade **eFigure 2.** Regression Estimates of Differences in Reading Scores by Gestational Age (Calendar Measure) and Grade

Supplement 2.
Data Sharing Statement
