# Supplementary material for: Association Between Gestational Age and Academic Achievement of Children Born at Term
Source: JAMA Netw Open. 2023 Jul 31;6(7):e2326451. doi: 10.1001/jamanetworkopen.2023.26451 (PMC10391305; doi:10.1001/jamanetworkopen.2023.26451)
Supplement: Supplement 1. — eFigure 1. Regression Estimates of Differences in Math Scores by Gestational Age (Calendar Measure) and Grade eFigure 2. Regression Estimates of Differences in Reading Scores by Gestational Age (Calendar Measure) and Grade [file jamanetwopen-e2326451-s001.pdf]

Supplementary Online Content

Wehby GL. Association between gestational age and academic achievement of children born at term. *JAMA Netw Open.* 2023;6(7):e2326451.
doi:10.1001/jamanetworkopen.2023.26451

eFigure 1. Regression Estimates of Differences in Math Scores by Gestational Age (Calendar Measure) and Grade

eFigure 2. Regression Estimates of Differences in Reading Scores by Gestational Age (Calendar Measure) and Grade

This supplementary material has been provided by the authors to give readers additional information about their work.

© 2023 Wehby GL. *JAMA Network Open.*

eFigure 1. Regression Estimates of Differences in Math Scores by Gestational Age (Calendar Measure) and Grade

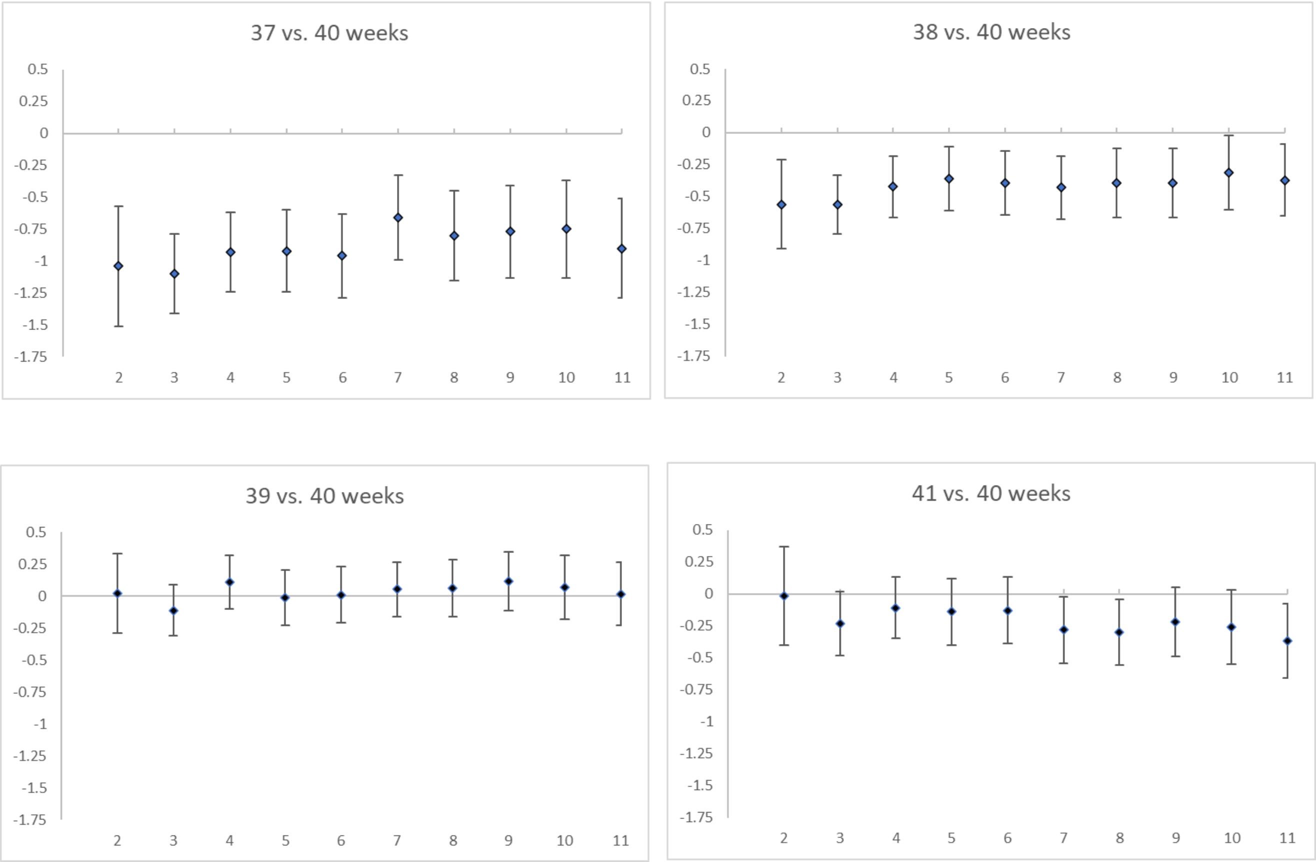

Notes: The point estimates are from a separate regression for each grade that estimates the differences in math scores across gestational age groups (40 weeks as the reference group). The regression adjusts for all covariates including labor induction and cesarean delivery. The differences in test scores are in national percentile rankings. 95% confidence intervals are shown around the point estimates.

© 2023 Wehby GL. *JAMA Network Open.*

eFigure 2. Regression Estimates of Differences in Reading Scores by Gestational Age (Calendar Measure) and Grade

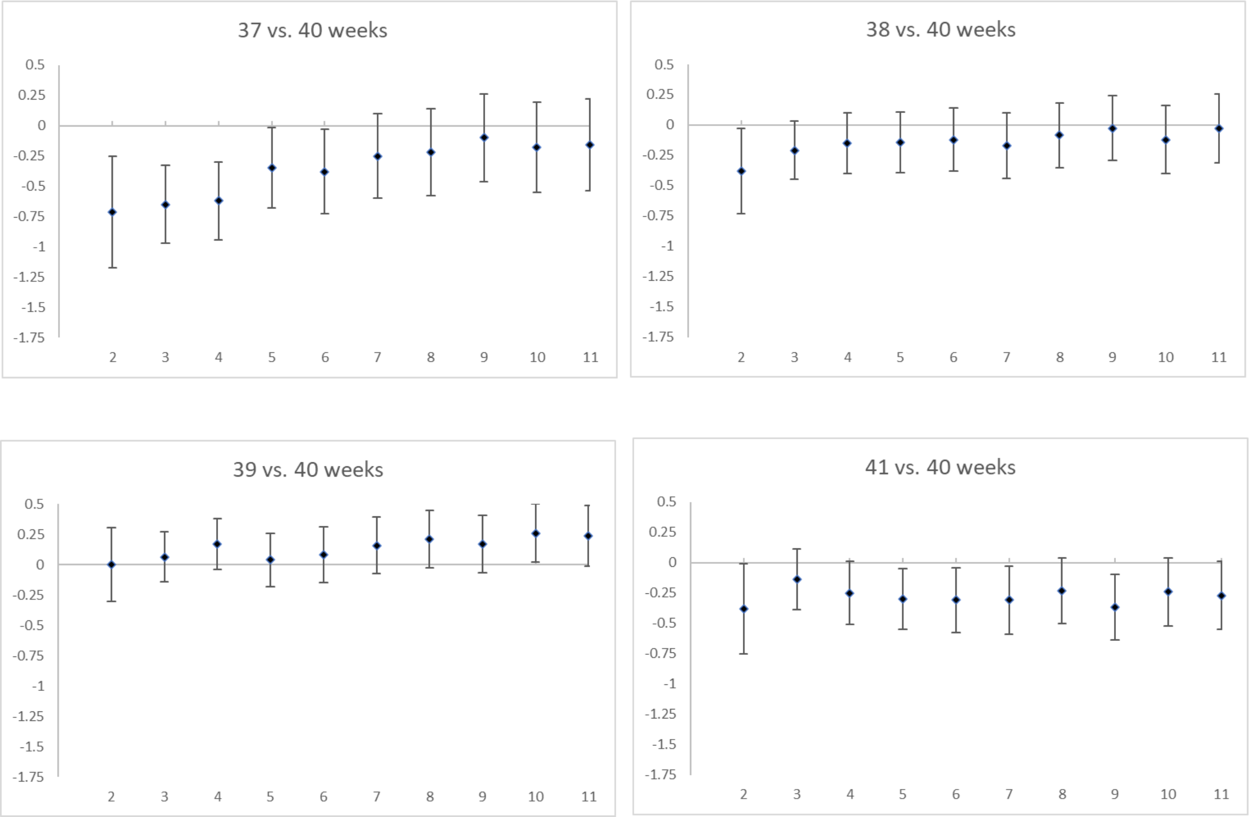

Notes: The point estimates are from a separate regression for each grade that estimates the differences in reading scores across gestational age groups (40 weeks as the reference group). The regression adjusts for all covariates including labor induction and cesarean delivery. The differences in test scores are in national percentile rankings. 95% confidence intervals are shown around the point estimates.

© 2023 Wehby GL. *JAMA Network Open.*
