# Supplementary material for: Association Between Gestational Age and Academic Achievement of Children Born at Term
Source: JAMA Netw Open. 2023 Jul 31;6(7):e2326451. doi: 10.1001/jamanetworkopen.2023.26451 (PMC10391305; doi:10.1001/jamanetworkopen.2023.26451)
Supplement: Supplement 2. — Data Sharing Statement [file jamanetwopen-e2326451-s002.pdf]

Data Sharing Statement

Wehby. Association Between Gestational Age and Academic Achievement of Children Born at Term. *JAMA Netw Open*. Published July 31, 2023. doi:10.1001/jamanetworkopen.2023.26451

Data
Data available: No

Additional Information
Explanation for why data not available: The study uses restricted-access data requiring DUAs with each researcher accessing the data.
